# Pulmonary Artery Pressure-Guided Telemonitoring Reduced Pulmonary Artery Pressure but Did Not Result in Higher Doses of Guideline-Directed Medical Therapy–Observations from an Advanced Elderly German Heart Failure Cohort

**DOI:** 10.3390/life12050766

**Published:** 2022-05-21

**Authors:** Ester J. Herrmann, Badrinarayanan Raghavan, Nina Eissing, Stephan Fichtlscherer, Christian W. Hamm, Birgit Assmus

**Affiliations:** 1 Department of Medicine I, Cardiology, University Hospital Giessen and Marburg, 35392 Giessen, Germany; ester.herrmann@innere.med.uni-giessen.de (E.J.H.); badrinarayanan.raghavan@innere.med.uni-giessen.de (B.R.); christian.hamm@innere.med.uni-giessen.de (C.W.H.); 2 Department of Medicine III, Cardiology, University Hospital Frankfurt am Main, 60590 Frankfurt am Main, Germany; ninas@eissing.net (N.E.); fichtlscherer@em.uni-frankfurt.de (S.F.); 3 German Center for Cardiovascular Research, DZHK, 10115 Berlin, Germany

**Keywords:** chronic heart failure, older patients, remote monitoring, pulmonary artery pressure, uptitration, guideline-directed medical therapy

## Abstract

**Introduction:** Remote pulmonary artery pressure (PAP)-guided heart failure (HF) therapy for NYHA class III patients has been shown to reduce hospitalizations and increase survival. We aimed to assess whether PAP monitoring allows for the increase in HF directed medication in an elderly German cohort of advanced HF patients already receiving clinically optimized HF medication. **Methods:** We analyzed PAP and HF medication dosage, including diuretics, in 24 patients (mean age, 76 years) using implanted PAP-sensors during the first 12 months of PAP-guided HF care in an interdisciplinary HF unit. **Results:** During 12 months of PAP-guided HF therapy, PAP decreased significantly (△PAP systolic–6 ± 10, △PAP diastolic–4 ± 7, △PAP mean–4 ± 8 mm Hg, *p* < 0.01 for all). 16% of patients had an unplanned HF hospitalization. There was no significant change over time with respect to the dosage of RAAS inhibitors (ACE-I/ARB/ARNI), Beta blockers, or MRA treatments. In contrast, the dosage of loop diuretics increased significantly (2.1 ± 0.5-fold) over time. In the comparison of a “responder” (patients with PAP and diuretic dose decline) and “non-responder” (patients with PAP and diuretic dose increase) group, there were no significant differences between any of the baseline, medication, or HF hospitalization characteristics between the two groups. **Conclusions:** In elderly patients treated with clinically optimized HF medication, no further evidence-based medication increase could be achieved using PAP-guided HF care. However, by individual adjustment of diuretic dosage, a significant decline in PAP over time occurred, which could not be predicted by any of the baseline characteristics.

## 1. Introduction

With 15 million patients, chronic heart failure (HF) HF is a very common disease in Europe and is the most frequent reason for a hospitalization due to illness. Because of progressive aging in Western societies, the prevalence increases steadily and leads to a high financial burden [1,2,3,4,5]. Symptoms of HF include reduced exercise tolerance, which is associated with increased (left ventricular) filling pressure with the passive backward transmission of elevated pressure into the pulmonary vasculature [6,7]. Cardiopulmonary congestion leads to an increased pulmonary arterial pressure (PAP). Diuretics are recommended to reduce the signs/symptoms of congestion. The quality of evidence regarding diuretics is poor, and their effects on morbidity and mortality have not been studied in randomized controlled trials (RCTs) [6], but the treatment with diuretics is mandatory to reduce hospitalizations [8]. Standard HF medications, such as beta blockers, angiotensin-converting enzyme inhibitors (ACE-I), angiotensin receptor blockers (ARB), aldosterone antagonists, angiotensin receptor-neprilysin inhibitors (ARNI), and sodium-glucose co-transporter 2 inhibitors (SGLT2 inhibitors) all improved survival [9,10,11,12,13,14,15] rates, prevented recurrent hospitalizations due to worsening HF, and improved the clinical status, functional capacity, and quality of life [6]. However, the target dose of evidence-based medication is generally difficult to reach outside of clinical trials [16], and elderly patients are even less likely to receive evidence-based HF therapy [17]. Previous analyses have shown that prevalent comorbidities and associated multi-drug treatments are the most prominent factors limiting uptitration in elderly people [18].

During the last 15 years, with the development of a battery-free sensor for the wireless transmission of PAP values by remote access from the patient’s home [19], clinical trials have demonstrated that ambulatory guidance of HF treatment using PA-pressure values resulted in reduced numbers of HF-related hospitalizations and improvement in NYHA class and quality of life [20].

The most detailed medication analysis so far comes from the randomized CHAMPION trial [21], where the analysis of medication changes revealed that diuretics were changed most frequently from all drug classes, and significantly more often in the PAP-treatment group compared to the control group. The daily diuretic dose increased significantly within 6 months in both the control and PAP-guided treatment group, whereas doses of neurohormonal antagonists, such as ACE-I or ARB, beta blockers, and aldosterone receptor antagonists increased significantly by 4 mg, enalapril-equivalent, 3 mg, carvedilol-equivalent, and 4 mg, spironolactone-equivalent in the PAP-guided treatment group, but not in the control group. In the control and intervention group, guideline-recommended doses could not be achieved for any of the medications.

The aim of the present early single center study was to assess whether in advanced elderly HF patients with already clinically optimized medical HF therapy at a tertiary care center in Germany, a further increase in evidence-based HF medication is possible with the support of PAP hemodynamic information during a period of 12 months. 

## 2. Methods

Patients in the HF outpatient department, with chronic HF in functional NYHA class III, individually optimized HF medication, and a cardiac decompensation within the last 12 months, were offered implantation of the PAP sensor CardioMEMS™ and participation in a single center telemonitoring registry. A total of 31 patients received implantation of a CardioMEMS™ sensor via right heart catheterization between 2015 and 2017 and were repeatedly trained in HF care by a European Society of Cardiology-certified HF nurse. However, only 30 patients were compliant after implantation and regularly (≥5 times per week) transmitted their data by 12 a.m. each day within 12 months of follow-up, or until initiation of palliative care or death. A total of 24 patients survived 12 months of follow-up and thus represent the present analysis cohort. 

### 2.1. Hemodynamic Telemonitoring and Follow-Up

Regular telephone contact was initiated by HF nurses with all patients, using a standardized questionnaire [adopted from the INH (Interdisciplinary Network for HF) trial] [22]. The nurses were supported by an HF specialist. According to the telemedical standard operating procedure, weekly telephone contacts were scheduled in the first 4 weeks after implantation, followed by contacts every two weeks from week 5 to week 8 after implantation, and a routine call every 4 weeks from week 9 onward, if no alerts occurred due to the crossing of the individually adapted optimal PAP target area. We applied the hemodynamic-guided HF management previously used in the CHAMPION trial [23]. In brief, medication was adjusted with the aim to maintain the diastolic PAP in the range of 8–15 mmHg (optivolemic state), with the goal to first increase guidelines-directed medical therapies (GDMT) up to the guideline recommended doses before the adjustment of diuretics. If the patients showed signs of low perfusion, the reduction of diuretics, fluid repletion, and/or the administration of inotropic agents were considered. Pressure measurements were reviewed at least weekly in the case of optivolemia and two to three times per week if diastolic PAP was outside the individually defined optimal range. Regular outpatient visits were scheduled every 3 months in the HF outpatient department.

For comparison of the diuretic dose, torasemide was converted into a furosemide equivalent by assuming a ratio of 1:4. All neurohormonal antagonist dose changes, which were collected via telephone contact or during office visits, were transformed into % of guideline recommended dose.

All patients provided written informed consent for participation in the single center registry (NCT03020043). The study complies with the Declaration of Helsinki and was approved by the local ethics committee.

### 2.2. Statistics

This study is a retrospective analysis of the first German experience with the hemodynamic-guided HF care in an advanced HF cohort of a university hospital. Patient baseline characteristics were calculated as mean and standard deviation. Dose changes for guideline-directed evidence-based HF medication and changes in PAP values at baseline and follow-up (6 and 12 months after the implant) were compared using a one-way ANOVA; for △PAP, a two-way ANOVA was used. Dose changes for loop diuretics and functional NYHA class at baseline and follow-up values (6 and 12 months after the implant) were compared using a paired *t*-test. A Spearman’s statistical correlation analysis was performed for changes in loop diuretic dose and PAP diastolic values. Statistical significance was assumed if *p* < 0.05, and all reported *p* values are two-sided. All the statistical analyses, calculations, and graphs were performed using GraphPad Prism software (version 9.0) for MS Windows (GraphPad Software, San Diego, CA, USA).

## 3. Results

The patient baseline characteristics in Table 1 show an elderly advanced chronic HF cohort (n = 24), mean age of 76 ± 8 years, predominantly male, and except for two patients, all other patients suffered from HF with reduced ejection fraction (HFrEF). The other two patients had a current LVEF of 40% with a history of a previous LVEF < 40%, so they were included in the medication analysis. 

N-terminal pro brain natriuretic peptide (NT-proBNP) values were elevated, and kidney function was moderately impaired in most patients. All patients received hemodynamic telemonitoring with the CardioMEMS™ system for severe NYHA class III HF. Only 21 of 24 patients were tolerating the RAAS blockade: one patient suffered from angioedema and two patients were intolerant of any RAAS inhibition due to hypotension, in addition to high potassium levels in one of these patients. A total of 92% of the patients took beta blockers, and 75% were on MRA (Table 2). 

Overall, systolic blood pressure was within the normal range, despite advanced HF, but the baseline PA pressure (PAP) was significantly elevated. Hemodynamic analysis showed that a total of 17 patients suffered from pulmonary hypertension (defined by mean PAP ≥ 25 mm Hg) [24]. Further classification revealed that n = 6 patients had isolated postcapillary pulmonary hypertension, one patient could not be classified, and 10 patients suffered from predominately combined pre- and postcapillary pulmonary hypertension (Table 3). 

**Figure 1:**
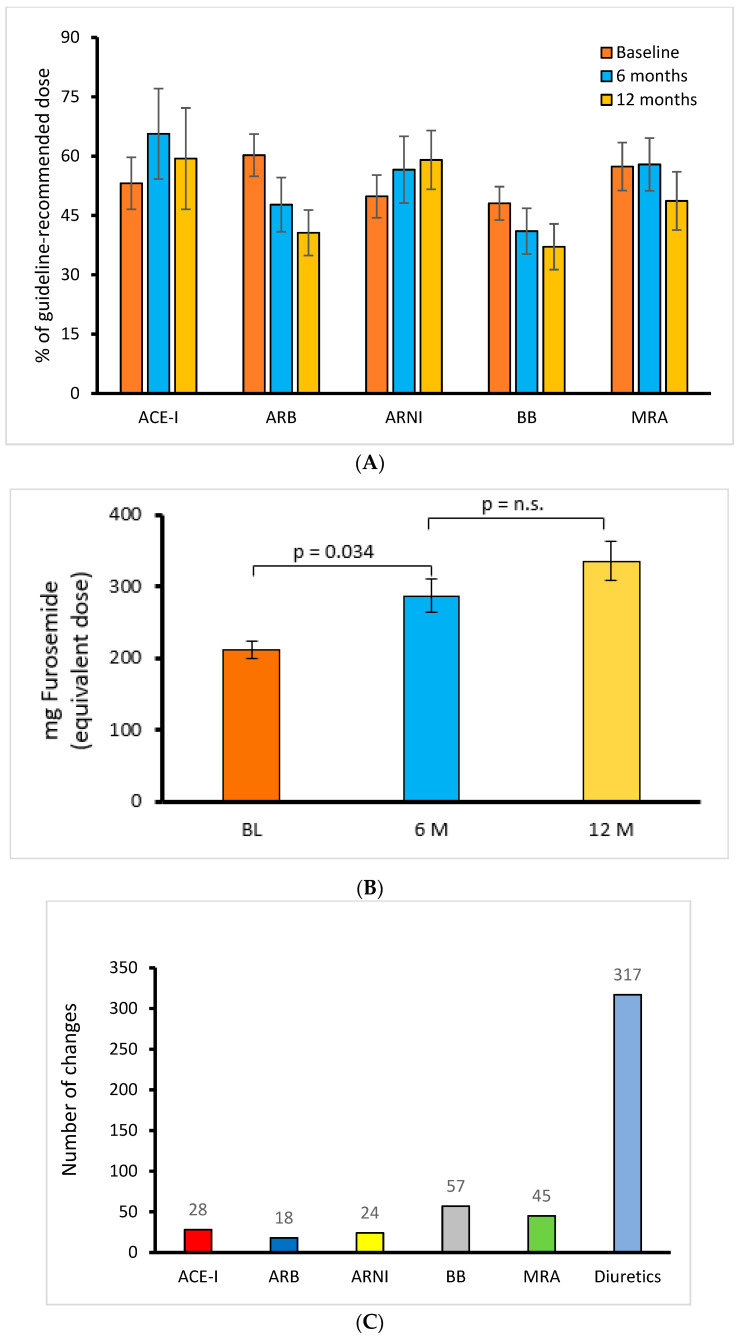

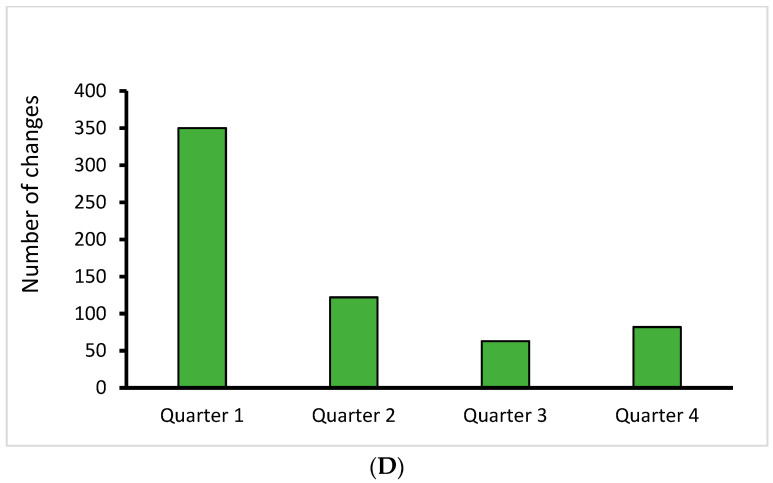
(**A**) Course of guideline-recommended doses of HF medication during 12 months of follow-up (all n.s.). (**B**) Mean loop diuretic dose at baseline, 6 months, and 12 months follow-up, calculated as the furosemide-equivalent dose. (**C**) Cumulative number of medication changes during 12 months of follow-up (total n = 513). (**D**) Cumulative medication changes per quarter during 12 months of follow-up.

At 12 months follow-up, there was no significant change in the neurohormonal blockade dose, when compared to baseline medication (Figure 1A).

Between 2015 and 2017, ARNI was introduced into HF therapy; thus, we observed increases in ARNI initiation and uptitration, with a parallel decline in ACEI and ARB. However, none of these changes were significant. In contrast, the loop diuretic dose increased significantly from the baseline to 6 months of follow-up (*p* = 0.034) and increased further until 12 months of follow-up (Figure 1B). 

As expected, diuretics were the drugs that were adjusted most frequently, as already shown in previous trials (n = 317 changes within 12 months), followed by ARNI/ACE-I/ARB (n = 24/28/18), BB (n = 57 changes), and MRA (n = 45 changes (Figure 1C)).

Most medication changes occurred within the first 3 months after implantation, demonstrating the efforts to uptitrate medication and achieve optimized diastolic PAP (Figure 1D). 

**Figure 2:**
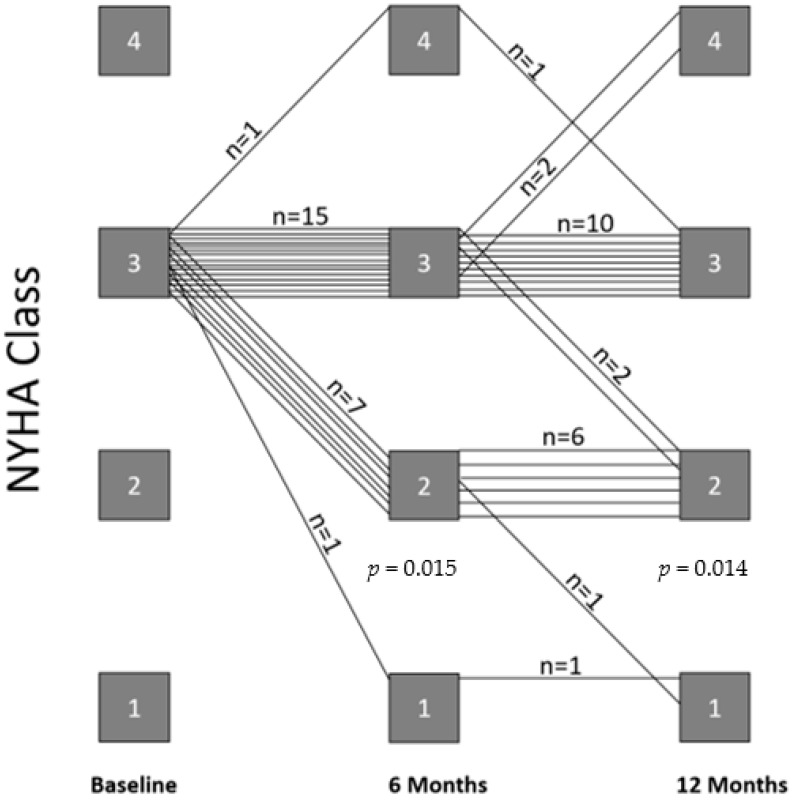
Changes in NYHA class within 12 months of PAP-guided HF therapy.

During the 12 months of PAP-guided HF management, we observed a statistically significant improvement in functional NYHA class from baseline to 6 months (*p* = 0.015) and up to 12 months of follow-up (*p* = 0.014; Figure 2). 

**Figure 3:**
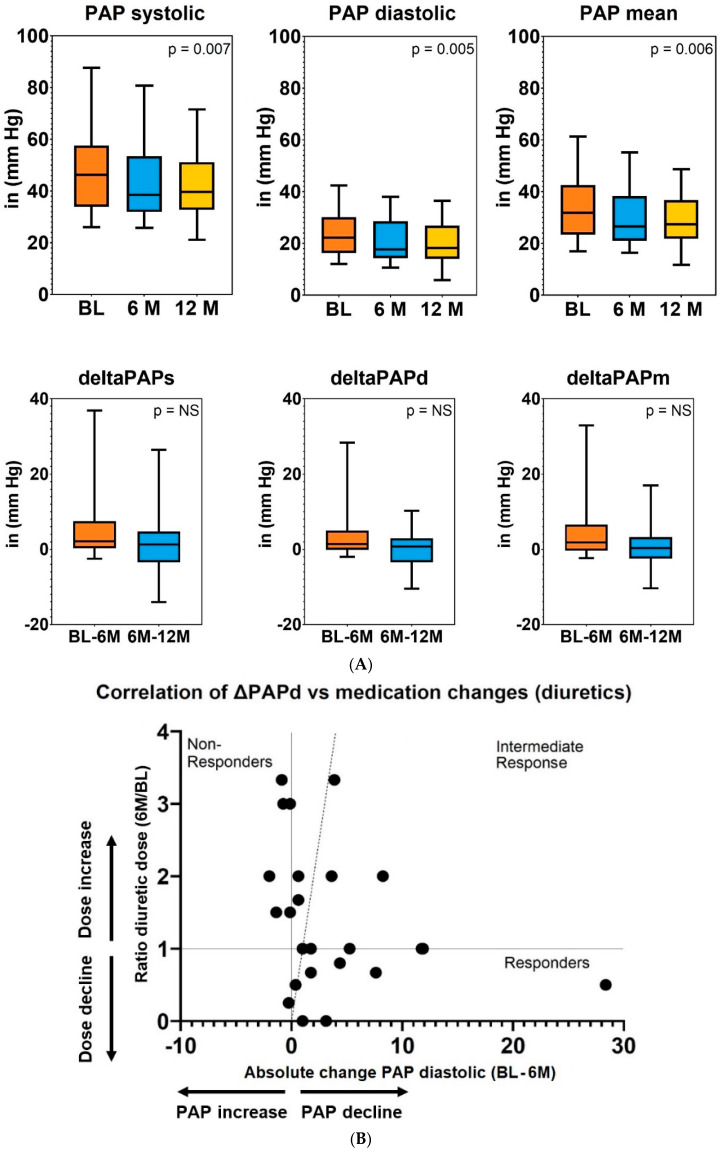

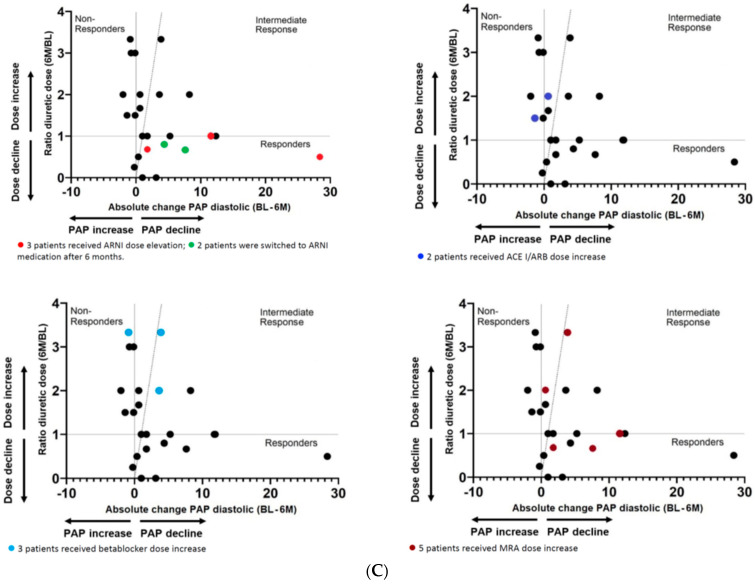
(**A**) Time course and absolute changes in PAP values during 12 months of PAP-guided HF therapy. (**B**) Association between changes in loop diuretic dose and diastolic PAP at 6 months. (**C**) Association between changes in loop diuretic dose (decline or increase) and diastolic PAP (decline or increase) at 6 months in patients receiving uptitration of different HF medication at 6 months.

In line with these finding, all PAP values decreased significantly, with an absolute reduction of systolic PA pressure of 6 mmHg (*p* = 0.007) and an absolute decline in diastolic PA-pressure of 4 mmHg (*p* = 0.005; Figure 3A). 

In order to assess whether the change in the loop diuretic dose is associated with a reduction in PAP, we correlated the change in the loop diuretic dose with the change of diastolic PAP. However, the change of diastolic PAP did not correlate with the change in the loop diuretic dose at 6 months (Figure 3B), nor at 12 months (data not shown). In addition, we could not identify any baseline clinical, hemodynamic, or laboratory characteristic that predicted the change in PAP or whether a patient could be classified as a “treatment responder,” defined as a patient with a PAP and diuretic dosage decrease, or a “non-responder,” defined as a patient with a PAP increase, despite diuretic increase. 

With respect to further medication modifications, all patients who were initiated or uptitrated with ARNI were in the responder group (Figure 3C), whereas the other changes of HF medication could not be correlated with changes in PAP or loop diuretic dose.

## 4. Discussion

The present study is a descriptive report about daily non-invasive hemodynamic monitoring with the CardioMEMS™ system in elderly patients in the German healthcare system with advanced chronic HF, correlating the changes of guideline-recommended HF medication doses during 12 months of follow-up with PAP changes. Our analysis has two main results: First, it demonstrates that no significant changes in guideline-directed medical therapies (GDMT) were possible with PAP-guided monitoring in advanced elderly HF patients already receiving clinically optimized HF-medication. According to the inclusion criteria of our registry, in close association with the CHAMPION trial [23], the PAP sensor system has been implanted into patients who already received drug and device treatments for HF [8]. Therefore, only minor changes were expected, although it was a prespecified aim of our registry to use PAP data for further individual uptitration of HF medication. 

Second, as expected, the average dosage of ACE-I and ARB declined over time, whereas ARNI dose and frequency increased non-significantly. This is due to the stepwise integration of ARNI since 2016, which is within the observation time of the present analysis. In a previously published report of the MEMS-HF trial, ARNI use was associated with less use of loop-diuretics compared to non-ARNI use at baseline [25]. In addition, during 9 months of follow-up, the loop diuretic dose increased to a non-significant, smaller extent in ARNI users compared to non-ARNI users [25]. In our single center cohort, loop diuretic dose was not significantly different between ARNI users and non-ARNI users, although the 5 patients who were either switched to ARNI or received ARNI dose increases demonstrated a decline in the loop diuretic dose, in addition to a decrease in PAP. Interestingly, the dose increases for other HF medications, such as ACE-I/ARB, beta blockers, or MRAs, did not show a uniform response for diuretic dose or PAP. 

With respect to other HF medication, only 50% of the recommended dose of MRA could be achieved in 16 HF patients, whereas 2 patients could not even tolerate an MRA. In 16 patients, a non-significant increase in MRA dose could be detected from baseline to 6 months, but this was followed by a subsequent decline until 12 months of follow-up due to recurrent hyperkalemia episodes. Currently, one would expect that improved results with a higher percentage of MRA treatment and doses can be expected, because SGLT2 inhibitors reduce the risk of MRA-associated hyperkaliemia [26], and novel potassium binders like patiromer and sodium zirconium cyclosilicate are now available [27,28] and are recommended in the ESC 2021 HF guidelines [6].

Most medication changes occurred within the first 3 months, and the frequency of changes decreased during 12 months of follow-up. This is in line with previous studies (CHAMPION and MEMS-HF) [21,25], which both showed that the treatment modifications occur with a great intensity right after PAP sensor implantation. However, the absolute number of medication changes per patient at 3 months was approximately 5.5 in CHAMPION (treatment group) and 3.2 in MEMS-HF, compared to 14.5 changes per patient within the first 3 months in the present cohort, which declined to 5.1 changes per patient over the following 3 months. This difference may be because the present patients were older and presented with slightly higher PAP and PVR at baseline, which may reflect more advanced disease. These efforts to optimize PAP with medication modification demonstrate the workload associated with hemodynamic-guided HF care in advanced elderly patients, which is critical for the estimation of HF nurse capacity within a telemedical center [29].

The drug class of SGLT-2-inhibitors were not included as antidiabetic nor HF therapy because patients were included into the registry in parallel to the publication of the EMPA-REG-OUTCOME trial [30] and prior to the updated guideline recommendation for antidiabetic treatment, 2018 [31]. More recently, the EMBRACE-HF randomized trial demonstrated that, in HFrEF patients with an implanted PA pressure sensor, Empagliflozin was associated with rapid reductions in PA pressures that were amplified over time and appeared to be independent of loop diuretic management [32]. However, this was not associated with improved quality of life. 

Finally, our results show that a significant reduction in PAP values could be achieved, despite the fact that we could not increase evidence-based HF medication in individually optimized NYHA class III elderly HF patients. The reduction in PAP was not directly correlated with diuretic or other medication dosage, suggesting that the decline in PAP values may be due to better adherence to HF care. 

## 5. Limitations

The present single center registry comprises only 24 patients with hemodynamic telemonitoring, with analyses of the changes of evidence-based HF medication during the 12 months of follow-up. The limited patient number is most likely responsible for our inability to identify a clear patient profile associated with a positive response to hemodynamic-guided heart failure therapy. However, the study represents a careful individual analysis of advanced HF patients being treated in a university outpatient setting. 

## Figures and Tables

**Table 1:** Baseline characteristics of n = 24 HF-patients.

**Age (in years)**	76 ± 8
**Gender—male; female (n)**	19; 5
**NYHA class**	3
**Diabetes mellitus (%)**	52
**HFmrEF (n)**	2
**HFrEF (n)**	22
**ICD/CRT-D/CRT-P/PM (n)**	12/6/1/3
**NT-proBNP (pg/mL)**	2810 ± 2809 (median 1755)
**Creatinine (mg/dl)**	1.5 ± 0.4

**Table 2:** Medication at baseline.

	n = 24	% of Guideline Recommended Dose (n = 24)
**ACE-Inhibitor (ACE-I); n (%)**	11 (46)	53 ± 25%
**Angiotensin-receptor blocker (ARB); n (%)**	4 (17)	55 + 25%
**Angiotensin receptor blocker** **Neprilysin inhibitor (ARNI); n (%)**	6 (25)	34 ± 50%
**Beta blocker (BB); n (%)**	22 (92)	42 ± 12.5%
**Mineralocorticoid-receptor** **antagonist (MRA); n (%)**	18 (75)	51 ± 50%
**Loop diuretics; n (%)**	22 (92)	-

**Table 3:** Hemodynamic baseline parameters.

**Systolic Blood Pressure (mmHg)**	116 ± 19
**PAP systolic (mmHg)**	48 ± 16
**PAP mean (mmHg)**	33 ± 12
**PAP diastolic (mmHg)**	24 ± 9
**PCWP (mmHg)**	17 ± 8
PVR (dyn*s/cm^5^)	317 ± 266

## Data Availability

The data are combined together and analyzed within the single center registry (NCT03020043) and can be obtained by written request.

## References

[1] Krishnan D.K., Pawlaczyk B., McCullough P.A., Enright S., Kunadi A., Vanhecke T.E. (2016). Point-of-Care, Ultraportable Echocardiography Predicts Diuretic Response in Patients Admitted with Acute Decompensated Heart Failure. Clin. Med. Insights. Cardiol..

[2] Herrmann E., Ecke A., Herrmann E., Eissing N., Fichtlscherer S., Zeiher A.M., Assmus B. (2018). Daily non-invasive haemodynamic telemonitoring for efficacy evaluation of MitraClip^®^ implantation in patients with advanced systolic heart failure. ESC Heart Fail. J..

[3] Casu G., Merella P. (2015). Diuretic Therapy in Heart Failure—Current Approaches. Eur. Cardiol..

[4] Christ M., Störk S., Dörr M., Heppner H.J., Müller C., Wachter R., Riemer U. (2016). Heart failure epidemiology 2000-2013: Insights from the German Federal Health Monitoring System. Eur. J. Heart Fail..

[5] Störk S., Handrock R., Jacob J., Walker J., Calado F., Lahoz R., Hupfer S., Klebs S. (2017). Epidemiology of heart failure in Germany: A retrospective database study. Clin. Res. Cardiol..

[6] McDonagh T.A., Metra M., Adamo M., Gardner R.S., Baumbach A., Böhm M., Burri H., Butler J., Čelutkienė J., Chioncel O. (2021). 2021 ESC Guidelines for the diagnosis and treatment of acute and chronic heart failure. Eur. Heart J..

[7] Rosenkranz S., Gibbs J.S.R., Wachter R., De Marco T., Vonk-Noordegraaf A., Vachiéry J.L. (2016). Left ventricular heart failure and pulmonary hypertension. Eur. Heart J..

[8] Ponikowski P., Voors A.A., Anker S.D., Bueno H., Cleland J.G.F., Coats A.J.S., Falk V., González-Juanatey J.R., Harjola V.-P., Jankowska E.A. (2016). 2016 ESC Guidelines for the diagnosis and treatment of acute and chronic heart failure: The Task Force for the diagnosis and treatment of acute and chronic heart failure of the European Society of Cardiology (ESC)Developed with the special contribution of the Heart Failure Association (HFA) of the ESC. Eur. Heart J..

[9] Packer M., Fowler M.B., Roecker E.B., Coats A.J.S., Katus H.A., Krum H., Mohacsi P., Rouleau J.L., Tendera M., Staiger C. (2002). Effect of carvedilol on the morbidity of patients with severe chronic heart failure: Results of the carvedilol prospective randomized cumulative survival (COPERNICUS) study. Circulation.

[10] Flather M.D., Shibata M.C., Coats A.J.S., Van Veldhuisen D.J., Parkhomenko A., Borbola J., Cohen-Solal A., Dumitrascu D., Ferrari R., Lechat P. (2005). Randomized trial to determine the effect of nebivolol on mortality and cardiovascular hospital admission in elderly patients with heart failure (SENIORS). Eur. Heart J..

[11] Khan M.S., Fonarow G.C., Ahmed A., Greene S.J., Vaduganathan M., Khan H., Marti C., Gheorghiade M., Butler J. (2017). Dose of Angiotensin-Converting Enzyme Inhibitors and Angiotensin Receptor Blockers and Outcomes in Heart Failure: A Meta-Analysis. Circ. Heart Fail..

[12] Zannad F., McMurray J.J.V., Krum H., van Veldhuisen D.J., Swedberg K., Shi H., Vincent J., Pocock S.J., Pitt B. (2011). Eplerenone in patients with systolic heart failure and mild symptoms. N. Engl. J. Med..

[13] McMurray J.J.V., Packer M., Desai A.S., Gong J., Lefkowitz M.P., Rizkala A.R., Rouleau J.L., Shi V.C., Solomon S.D., Swedberg K. (2014). Angiotensin-neprilysin inhibition versus enalapril in heart failure. N. Engl. J. Med..

[14] McMurray J.J.V., Solomon S.D., Inzucchi S.E., Køber L., Kosiborod M.N., Martinez F.A., Ponikowski P., Sabatine M.S., Anand I.S., Bělohlávek J. (2019). Dapagliflozin in Patients with Heart Failure and Reduced Ejection Fraction. N. Engl. J. Med..

[15] Packer M., Anker S.D., Butler J., Filippatos G., Pocock S.J., Carson P., Januzzi J., Verma S., Tsutsui H., Brueckmann M. (2020). Cardiovascular and Renal Outcomes with Empagliflozin in Heart Failure. N. Engl. J. Med..

[16] Ouwerkerk W., Voors A.A., Anker S.D., Cleland J.G., Dickstein K., Filippatos G., van der Harst P., Hillege H.L., Lang C.C., Ter Maaten J.M. (2017). Determinants and clinical outcome of uptitration of ACE-inhibitors and beta-blockers in patients with heart failure: A prospective European study. Eur. Heart J..

[17] Cowie M.R., Flett A., Cowburn P., Foley P., Chandrasekaran B., Loke I., Critoph C., Gardner R.S., Guha K., Betts T.R. (2022). Real-world evidence in a national health service: Results of the UK CardioMEMS HF System Post-Market Study. ESC Heart Fail. J..

[18] Stolfo D., Lund L.H., Becher P.M., Orsini N., Thorvaldsen T., Benson L., Hage C., Dahlström U., Sinagra G., Savarese G. (2022). Use of evidence-based therapy in heart failure with reduced ejection fraction across age strata. Eur. J. Heart Fail..

[19] Castro P.F., Concepción R., Bourge R.C., Martínez A., Alcaino M., Deck C., Ferrada M., Alfaro M., Perrone S. (2007). A wireless pressure sensor for monitoring pulmonary artery pressure in advanced heart failure: Initial experience. J. Heart Lung Transplant..

[20] Abraham W.T., Stevenson L.W., Bourge R.C., Lindenfeld J.A., Bauman J.G., Adamson P.B. (2016). Sustained efficacy of pulmonary artery pressure to guide adjustment of chronic heart failure therapy: Complete follow-up results from the CHAMPION randomised trial. Lancet.

[21] Costanzo M.R., Stevenson L.W., Adamson P.B., Desai A.S., Heywood J.T., Bourge R.C., Bauman J., Abraham W.T. (2016). Interventions Linked to Decreased Heart Failure Hospitalizations During Ambulatory Pulmonary Artery Pressure Monitoring. JACC Heart Fail..

[22] Angermann C.E., Störk S., Gelbrich G., Faller H., Jahns R., Frantz S., Loeffler M., Ertl G. (2012). Mode of action and effects of standardized collaborative disease management on mortality and morbidity in patients with systolic heart failure the interdisciplinary network for heart failure (INH) study. Circ. Heart Fail..

[23] Abraham W.T., Adamson P.B., Bourge R.C., Aaron M.F., Costanzo M.R., Stevenson L.W., Strickland W., Neelagaru S., Raval N., Krueger S. (2011). Wireless pulmonary artery haemodynamic monitoring in chronic heart failure: A randomised controlled trial. Lancet.

[24] Galiè N., Humbert M., Vachiery J.-L., Gibbs S., Lang I., Torbicki A., Simonneau G., Peacock A., Vonk Noordegraaf A., Beghetti M. (2016). 2015 ESC/ERS Guidelines for the diagnosis and treatment of pulmonary hypertension: The Joint Task Force for the Diagnosis and Treatment of Pulmonary Hypertension of the European Society of Cardiology (ESC) and the European Respiratory Society (ERS): Endo. Eur. Heart J..

[25] Böhm M., Assmus B., Anker S.D., Asselbergs F.W., Brachmann J., Brett M.-E., Brugts J.J., Ertl G., Wang A., Hilker L. (2021). Less loop diuretic use in patients on sacubitril/valsartan undergoing remote pulmonary artery pressure monitoring. ESC Heart Fail..

[26] Kristensen S.L., Docherty K.F., Jhund P.S., Bengtsson O., Demets D.L., Inzucchi S.E., Kober L., Kosiborod M.N., Langkilde A.M., Martinez F.A. (2020). Dapagliflozin reduces the risk of hyperkalaemia in patients with heart failure and reduced ejection fraction: A secondary analysis DAPA-HF. Eur. Heart J..

[27] Weir M.R., Bakris G.L., Bushinsky D.A., Mayo M.R., Garza D., Stasiv Y., Wittes J., Christ-Schmidt H., Berman L., Pitt B. (2015). Patiromer in patients with kidney disease and hyperkalemia receiving RAAS inhibitors. N. Engl. J. Med..

[28] Zannad F., Hsu B.-G., Maeda Y., Shin S.K., Vishneva E.M., Rensfeldt M., Eklund S., Zhao J. (2020). Efficacy and safety of sodium zirconium cyclosilicate for hyperkalaemia: The randomized, placebo-controlled HARMONIZE-Global study. ESC Heart Fail..

[29] Helms T.M., Perings C.A., Sommer P., Köhler F., Frey N., von Haehling S., Tiefenbacher C., Rybak K., Sack S., Stockburger M. (2022). Positionspapier zur Zertifizierung von Telemedizinzentren. Der Kardiol..

[30] Zinman B., Wanner C., Lachin J.M., Fitchett D., Bluhmki E., Hantel S., Mattheus M., Devins T., Johansen O.E., Woerle H.J. (2015). Empagliflozin, Cardiovascular Outcomes, and Mortality in Type 2 Diabetes. N. Engl. J. Med..

[31] Davies M.J., D’Alessio D.A., Fradkin J., Kernan W.N., Mathieu C., Mingrone G., Rossing P., Tsapas A., Wexler D.J., Buse J.B. (2018). Management of Hyperglycemia in Type 2 Diabetes, 2018. A Consensus Report by the American Diabetes Association (ADA) and the European Association for the Study of Diabetes (EASD). Diabetes Care.

[32] Nassif M.E., Qintar M., Windsor S.L., Jermyn R., Shavelle D.M., Tang F., Lamba S., Bhatt K., Brush J., Civitello A. (2021). Empagliflozin Effects on Pulmonary Artery Pressure in Patients with Heart Failure. Circulation.

